# Targeted CSF metabolomics and conformal prediction improve diagnostic accuracy of normal pressure hydrocephalus

**DOI:** 10.1186/s12987-026-00771-z

**Published:** 2026-02-07

**Authors:** Ulrika Hofling, Jenny Jakobsson, Ida Erngren, Oskar Ekman, Eva Freyhult, Akshai Parakkal Sreenivasan, Jakob Siljebo, Sylwia Libard, Lena Kilander, Malin Löwenmark, Martin Ingelsson, Kim Kultima, Johan Virhammar

**Affiliations:** 1 Department of Medical Sciences, Neurology, Uppsala University, Akademiska Sjukhuset, Ing 85, Uppsala, 751 85 Sweden; 2 Department of Medical Sciences, Clinical Chemistry, Uppsala University, Uppsala, Sweden; 3 Department of Surgical Sciences, Uppsala University, Uppsala, Sweden; 4 Department of Cell and Molecular Biology, National Bioinformatics Infrastructure Sweden, Science for Life Laboratory, Uppsala University, Uppsala, Sweden; 5 Department of Pathology, Uppsala University Hospital, Uppsala, Sweden; 6 Department of Immunology, Genetics and Pathology, Uppsala University, Uppsala, Sweden; 7 Department of Public Health and Caring Sciences, Clinical Geriatrics, Rudbeck Laboratory, Uppsala University, Uppsala, Sweden; 8 Krembil Brain Institute, University Health Network, Toronto, Ontario Canada; 9 Tanz Centre for Research in Neurodegenerative Diseases, Departments of Medicine and Laboratory Medicine & Pathobiology, University of Toronto, Toronto, Ontario Canada; 10 Department of Public Health and Caring Sciences, Molecular Geriatrics, Rudbeck Laboratory, Uppsala University, Uppsala, Sweden

**Keywords:** iNPH, CSF, Metabolomics, Biomarkers, LC-MS, Neurodegeneration, Oxidative stress, Glymphatic system

## Abstract

**Background and objectives:**

Idiopathic normal pressure hydrocephalus (iNPH) is a progressive but treatable neurological disorder. Yet, diagnosis is often confounded by overlapping symptoms and biomarker profiles with Alzheimer’s disease (AD), mild cognitive impairment (MCI), and frontotemporal dementia (FTD). We aimed to determine whether cerebrospinal fluid (CSF) metabolomic profiling, combined with uncertainty-aware machine learning using conformal prediction (CP), could improve diagnostic differentiation of iNPH.

**Methods:**

CSF samples were collected from 120 patients with iNPH, 44 healthy controls, and 152 individuals with AD, MCI, or FTD. Targeted metabolomics of 59 metabolites was performed using liquid chromatography–high-resolution mass spectrometry. Group differences were assessed using age- and sex-adjusted regression models. Multivariate classification with partial least squares discriminant analysis (PLS-DA) incorporated metabolites, demographics, and conventional biomarkers (amyloid-β42, tau, phosphorylated tau). CP was applied to address individual-level diagnostic uncertainty.

**Results:**

Eight metabolites (proline, threonine, histidine, tyrosine, tryptophan, isobutyrylcarnitine, citric acid, and dehydroascorbic acid) were consistently reduced in iNPH (q < 0.05), independent of ventricular volume and cortical tau or amyloid-β pathology. An integrated PLS-DA model combining metabolomic, demographic, and AD-biomarker data achieved excellent discrimination (AUC = 0.97). CP provided calibrated case-level confidence, identifying clear-cut and uncertain cases while maintaining high accuracy (94% for iNPH, 97% for not-iNPH).

**Discussion:**

iNPH exhibits a distinct CSF metabolomic signature reflecting altered amino acid metabolism, mitochondrial function, and oxidative stress. Integrating metabolomic data with established biomarkers enhances diagnostic accuracy, while CP adds individualized uncertainty estimates to improve diagnostic confidence and guide treatment decisions.

**Supplementary Information:**

The online version contains supplementary material available at 10.1186/s12987-026-00771-z.

## Introduction

Idiopathic normal pressure hydrocephalus (iNPH) is a progressive neurological disorder affecting older adults, clinically defined by the classic triad of gait disturbance, cognitive impairment, and urinary incontinence [1, 2]. Among these, gait abnormalities are typically the earliest and most prominent symptom [3]. Disruption of cerebrospinal fluid (CSF) dynamics is a central feature of the condition, and dysfunction of the glymphatic system is discussed as a contributing factor [2, 4].

Despite its distinct clinical presentation, the diagnosis and management of iNPH remains challenging, and often includes different invasive methods to manipulate or measure the dynamics of the CSF-system. Diagnosing iNPH can also be a clinical challenge due to symptom overlap with neurodegenerative conditions such as Alzheimer’s disease (AD), and the frequent coexistence of these pathologies in aging populations [4]. Some centers use CSF biomarkers, such as amyloid beta (amyloid-β42) and tau proteins, to improve diagnostic accuracy in the diagnostic work-up [5–7]. While these biomarkers help to identify AD pathology, they lack specificity for iNPH and are insufficient to differentiate between conditions.

Recent advances in CSF proteomic and metabolomic profiling have opened new avenues for exploring disease mechanisms and enhancing diagnostic precision. Preliminary studies comparing CSF metabolite signatures in iNPH and AD suggest metabolomics may offer valuable insights into differential diagnosis [8–11]. However, despite advances in biomarker discovery, significant limitations remain in clinical applications, including the inability to communicate uncertainty in individual predictions, unlike the aggregate measures of model performance typically reported (e.g., area under the curve, AUC), and the lack of explainability or interpretability at the level of a specific prediction [12].

To capture individual uncertainty, advanced computational approaches such as predictive models augmented with conformal prediction (CP) offer a robust framework for clinical decision-making [13–15]. CP enhances machine learning classifiers by assigning valid confidence measures to each prediction, thereby explicitly quantifying diagnostic uncertainty. In idiopathic normal pressure hydrocephalus (iNPH), where symptom overlap with AD and other dementias complicates diagnosis, CP can mitigate misclassification risk by flagging ambiguous cases and enabling more reliable differentiation. This uncertainty-aware approach is particularly valuable for identifying patients who may benefit from additional confirmatory testing.

The aim of this study was to identify novel biomarker patterns and metabolic signatures that extend beyond previously examined non-specific CSF markers, such as amyloid-β42 and tau, in order to distinguish iNPH from its major diagnostic mimics AD, AD-related mild cognitive impairment (ADMCI), stable MCI, and frontotemporal dementia (FTD). Furthermore, by incorporating CP into the analysis, we sought to provide individualized measures of diagnostic uncertainty, thereby enhancing the clinical interpretability of the results.

## Methods

### Participants

A total of 316 individuals were included in the study. Patients diagnosed with iNPH were recruited prospectively at Uppsala University Hospital between 2014 and 2020. All eligible participants with available CSF samples during the study period were included, no formal sample size calculation was performed. All participants underwent a comprehensive evaluation by a specialized NPH multidisciplinary team, with diagnosis determined according to international guidelines for iNPH [16]. Radiological data confirming normal ventricular size were not available for controls or other diagnostic groups.

Patients with AD, ADMCI, and MCI were diagnosed according to the National Institute on Aging and Alzheimer’s Association (NIA-AA) criteria. FTD diagnoses were determined using established clinical criteria combined with neuroimaging findings [17–19]. Diagnoses and lumbar punctures for participants in the neurodegenerative disease groups were performed between 2005 and 2018. Patients classified in the MCI group were longitudinally followed for 4–9 years following CSF sampling to ensure diagnostic stability. Importantly, none of these participants converted to AD dementia during the follow-up period, confirming their stable MCI classification.

The control group (C) consisted of neurologically healthy individuals without neurocognitive disorders, recruited at Uppsala University Hospital through local newspaper advertisements and from a cohort of older healthy individuals, explaining the higher median age. All analyses were adjusted for age to minimize bias. The study protocol received approval from the Swedish Ethical Review Authority. Written informed consent was obtained from all study participants, including patients and healthy controls, before enrollment and sample collection [20]. The sex and age distribution across all study groups is presented in Table 1.

**Table 1:** Demographic and biomarker characteristics across groups. Group differences were assessed using age- and sex-adjusted regression models. Values are presented as medians (Q1, Q3). In-group correlations between age and CSF biomarkers are provided in Supplementary table 1

Characteristics	iNPH (n = 120)	AD (n = 72)	ADMCI (n = 24)	FTD (n = 8)	MCI (n = 48)	Control (n = 44)
**Age**, median (Q1, Q3)	74 (70, 78)	72 (66, 76)	71 (67, 76)	71 (64, 76)	70 (61, 74)	88 (82, 88)
**Female**, n (%)	46 (38%)	43 (60%)	13 (54%)	3 (38%)	17 (35%)	10 (23%)
**Male**, n (%)	74 (62%)	29 (40%)	11 (46%)	5 (63%)	31 (65%)	34 (77%)
**Amyloid-β42**, median (Q1, Q3), ng/L	586 (418, 680)	400 (340, 465)	387 (350, 483)	710 (595, 819)	754 (645, 995)	663 (494, 927)
**Tau**, median (Q1, Q3), ng/L	180 (139, 240)	680 (471, 920)	528 (445, 650)	265 (222, 460)	244 (186, 330)	425 (353, 681)
**Ptau**, median (Q1, Q3), ng/L	26 (21, 32)	84 (60, 115)	77 (55, 93)	46 (35, 55)	45 (35, 54)	64 (45, 78)

*Abbreviations: AD: amyloid-β42: Amyloid-beta 42, tau: total tau, ptau: phosphorylated tau*

*Normal reference ranges for biomarkers: amyloid-β42: > 530 ng/L; tau: < 350 ng/L*

### Sample collection

CSF samples from all diagnostic groups and controls were obtained via lumbar puncture. All samples were stored in polypropylene tubes and frozen at − 70 °C. Before analysis, samples were thawed, aliquoted into microtubes, and refrozen at − 70 °C to maintain sample integrity. No adverse events related to CSF sampling or cortical biopsies were reported.

### Sample preparation

Thawed CSF samples were processed for targeted metabolomics using high-performance liquid chromatography coupled to high-resolution mass spectrometry (HPLC-HRMS), following a previously published protocol [21]. Analysts performing metabolomic profiling were blinded to clinical diagnoses and biopsy results. Briefly, 100 μL samples underwent protein precipitation with 300 μL cold methanol solution. Samples were vortexed for 15 seconds and incubated at − 20 °C for 1 hour. Following incubation, samples were centrifuged at 21,100 RCF at 4 °C for 15 minutes. A 300 μL aliquot of the supernatant was transferred to new tubes and concentrated by evaporation under a gentle nitrogen stream. The dried samples were reconstituted with 40 μL of 5% methanol in 95% H₂O, transferred into HPLC vials, and stored at − 80 °C until analysis.

### Mass spectrometry analysis

The HPLC-HRMS analysis was performed using an Ultimate 3000 HPLC system (Thermo Scientific, Waltham, MA, USA) coupled to a high-resolution hybrid quadrupole Q Exactive Orbitrap mass spectrometer (Thermo Scientific) operating in positive ionization mode with a target m/z range of 55–820. Quality control (QC) measures included pooled QC samples created by combining 10 μL aliquots from prepared samples to generate a global QC sample, which was repeatedly injected alongside blank samples throughout the analytical sequence.Sample analysis involved injecting five μL volumes using a method optimized for non-polar metabolites that employed reversed-phase liquid chromatography. Chromatographic separation was achieved using a C18 column (Accucore aQ RP C18 column, 100 × 2.1 mm, 2.6 μm, Thermo Scientific). A 22-minute gradient elution was employed with mobile phase A consisting of H₂O with 0.1% formic acid and mobile phase B comprising 90% acetonitrile and 10% 2-propanol with 0.1% formic acid. The mass spectrometer settings utilized have been previously described in detail [21].

### Ventricular volume

Ventricular volume was measured using two methods. Evans’ index was manually determined from axial sections of preoperative computed tomography scans [22]. In addition, in cases with an available preoperative 3DT1 MRI scan, ventricular volume was assessed with *SmartPaint*, a freely available, semi-automatic volumetric segmentation software [23].

### Brain biopsy procedures

Patients with iNPH were treated with insertion of a shunt system with the burr hole placed over the frontal lobe (most often the right side). When no surgical contraindications were present, one needle biopsy from the cortex measuring approximately 2 × 3 mm was obtained immediately before shunt insertion, as previously described [24]. The biopsy specimens underwent standard histopathological processing, including fixation in formalin solution and paraffin embedding, followed by hematoxylin-eosin staining and additional immunohistochemical methods [24]. Qualified neuropathologists performed diagnostic evaluation of the samples and assessed the presence or absence of hyperphosphorylated tau (Thermo Fisher Scientific; Clone Tau8) and amyloid-β (Dako-Agilent; Clone 6F/3D) protein aggregations within the biopsy specimens. Here we define biopsy findings of both tau and amyloid-β42 as AD+ and all other combinations as AD-.

### Statistical analysis

Raw metabolomics data were processed in TraceFinder (version 4.1, Thermo Scientific). Relative metabolite concentrations were normalized for weekly signal drift using a locally estimated scatterplot smoothing (LOESS) model, and inter-week effects were corrected by dividing each metabolite intensity by the weekly median global QC intensity. In total, 219 metabolites spanning diverse classes and pathways were analyzed, of which 59 (26.9%) met the quality control threshold (≥90% non-missing values) and were included in subsequent analyses.

Linear regression models were employed to find alterations in metabolite concentrations for the five patient groups and control subjects. All regression models were adjusted for age and sex to account for demographic differences between groups. In addition, a sensitivity analysis was performed using a subset of healthy controls closer in age to the iNPH group, which confirmed the main findings, although with slightly higher p-values due to smaller sample size. The linear regression models were evaluated using analysis of variance (ANOVA) F-tests, while pairwise group comparisons were conducted using post hoc t-statistics. The ANOVA p-values were adjusted for multiple testing using the Benjamini-Hochberg method for false discovery rate (FDR), with adjusted p-values (q values) below 5% considered statistically significant. Correlations between metabolite measurements were assessed using Pearson’s correlation, and *p* < 0.01 was considered significant. We also computed withingroup correlations between age and the clinically used CSF biomarkers amyloidβ42, tau, ptau for each diagnostic group (Supplementary Table S1).

A multivariate partial least-squares discriminant analysis (PLS-DA) model was constructed to distinguish iNPH from the other patient groups (AD, ADMCI, MCI, FTD) (controls excluded). Three different PLS-DA models were built. The first included age, sex, and the clinically used CSF biomarkers. The second included the 59 measured metabolites, and the third included age and sex, the established CSF biomarkers, and all metabolite data. Model performance was evaluated using ten five-fold cross-validations.

### Conformal prediction

Conformal prediction (CP) is a model-agnostic framework that can be applied to any machine learning algorithm to quantify prediction uncertainty [13]. CP was implemented as a post-hoc calibration layer on top of a supervised PLS-DA model. As the calibration step relies on labeled data to compute nonconformity scores and guarantee coverage, the resulting conformal predictions are supervised. In classification problems, CP generates a prediction set that may include one, several, or no class labels. For each potential label, CP computes a p-value indicating how unusual a new data point appears compared with the calibration data when assigned that label. A low p-value suggests that the data point differs substantially from previously observed examples of that label. In the transductive CP (TCP) framework, each new data point is evaluated by temporarily adding it to the dataset under each possible class label. Conformity scores are then recomputed to assess how well the data point fits relative to the calibration data. This process is repeated for every prediction, which makes TCP more computationally demanding but also particularly reliable in smaller datasets. For each label, a p-value is calculated as the proportion of calibration scores greater than or equal to the test data point score. Labels with p-values above the predefined significance level were retained in the final prediction set. Depending on the results, the prediction set could contain a single label (high confidence), multiple labels (ambiguous classification), or be empty (low confidence).

TCP was employed in this study due to its ability to maintain calibration of prediction regions under small-sample conditions. This calibration was assessed by comparing expected and observed error rates, with good calibration indicated by a diagonal relationship in the calibration plot.

## Results

### Study participants

The study cohort comprised participants with a median age of 74 years (44–89), with an even sex distribution of 42% women and 58% men. Established CSF biomarkers (amyloidβ42, tau, and ptau) analyzed as part of routine clinical work-up are presented in Table 1. Withingroup correlations between age and biomarkers were weak or nonsignificant in most groups (Supplementary Table S1), indicating that betweengroup biomarker differences are unlikely to be explained by age.

The iNPH group demonstrated significantly lower levels of tau and ptau compared to the other diagnostic groups and reduced levels of amyloid-β42 compared to healthy controls (Supplementary Figure 1). Principal component analysis (PCA) of the metabolic profiles demonstrated partial separation between patient groups, as shown in Supplementary Figure 2.

### Metabolomic differences between iNPH and neurodegenerative disorders

**Fig. 1:**
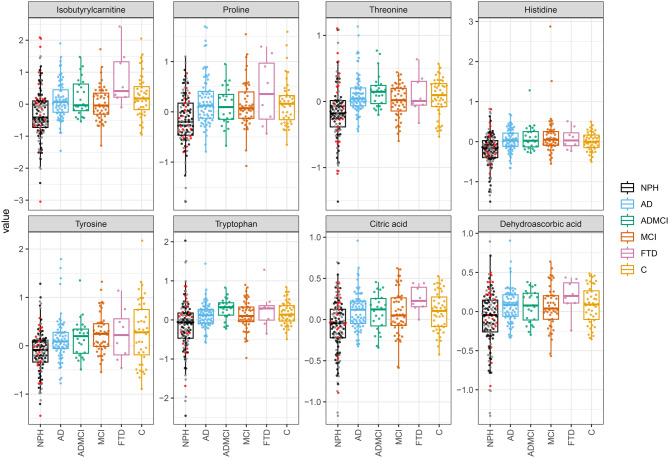
Box-plots of the eight metabolites that were lower in iNPH compared with controls and all other groups (significant global p-value (ANOVA; FDR 5%) and also *p* < 0.05 in the pairwise iNPH comparisons). For iNPH, the individuals with AD+ biopsies are red, AD- are black, and patients with no biopsy are grey

Fifty-nine metabolites were semi-quantified in the CSF of all the included groups (Supplementary Table 2). Of the 59 metabolites, eight demonstrated overall statistically significant differences between groups (ANOVA F-test with FDR correction at 5%) and showed consistent pairwise separation of iNPH from all other diagnostic groups and controls (*p* < 0.05; Table 2, Fig. 1). These discriminatory metabolites included five amino acids: proline, threonine, histidine, tyrosine, and tryptophan, and in addition, isobutyrylcarnitine, citric acid, and the vitamin derivative dehydroascorbic acid. Notably, all eight metabolites were detected at lower concentrations in the iNPH group than in healthy controls and the neurodegenerative disorder groups.

**Table 2:** Linear regression, adjusted for age and sex, was used to identify metabolites differing between NPH and other diagnostic groups. ANOVA F-tests with FDR correction determined overall significance, and pairwise comparisons are shown as log_2_ Fold changes (iNPH vs other group) with confidence intervals and p-values

Metabolite pANOVA (qANOVA) Log2FC [log2FC.low, log2FC.high], p	AD	ADMCI	MCI	FTD	C
Isobutyrylcarnitine 2,0E-07 (1,1E-05)	−0.48 [−0.69, −0.27], 1.2e–5	−0.55 [−0.86, −0.23], 6.8e–4	−0.37 [−0.62, −0.12], 0.003	−1.12 [−1.63, −0.61], 2.2e–5	−0.43 [−0.71, −0.15], 0.003
Proline 4,8E-07 (1,1E-05)	−0.40 [−0.55, −0.25], 2.9e–7	−0.29 [−0.51, −0.07], 0.010	−0.35 [−0.52, −0.17], 1.4e–4	−0.59 [−0.95, −0.23], 0.001	−0.28 [−0.48, −0.08], 0.007
Threonine 5,5E-07 (1,1E-05)	−0.28 [−0.38, −0.17], 3.8e–7	−0.32 [−0.48, −0.17], 5.4e–5	−0.17 [−0.29, −0.04], 0.009	−0.27 [−0.52, −0.01], 0.040	−0.21 [−0.35, −0.07], 0.003
Histidine 8,4E-07 (1,2E-05)	−0.24 [−0.36, −0.13], 5.3e–5	−0.30 [−0.47, −0.13], 5.6e–4	−0.34 [−0.48, −0.20], 1.5e–6	−0.28 [−0.56, −0.00], 0.050	−0.22 [−0.37, −0.06], 0.006
Tyrosine 1,0E-06 (2,3E-05)	−0.29 [−0.43, −0.15], 9.2e–5	−0.31 [−0.52, −0.10], 0.004	−0.42 [−0.59, −0.25], 1.6e–6	−0.39 [−0.73, −0.05], 0.026	−0.27 [−0.46, −0.08], 0.010
Tryptophan 2,5E-06 (2,4E-05)	−0.31 [−0.45, −0.16], 4.7e–5	−0.47 [−0.68, −0.25], 2.3e–4	−0.32 [−0.49, −0.15], 2.6e–4	−0.46 [−0.81, −0.11], 0.010	−0.23 [−0.42, −0.03], 0.022
Citric acid 3,2E-06 (2,7E-05)	−0.21 [−0.30, −0.12], 2.5e–6	−0.18 [−0.31, −0.06], 0.005	−0.16 [−0.26, −0.06], 0.002	−0.33 [−0.54, −0.13], 0.002	−0.16 [−0.27, −0.05], 0.006
Dehydroascorbic acid 7,0E-05 (4,3E-04)	−0.19 [−0.28, −0.10], 4.6e–5	−0.18 [−0.31, −0.04], 0.010	−0.15 [−0.26, −0.05], 0.005	−0.29 [−0.51, −0.07], 0.009	−0.16 [−0.28, −0.04], 0.008

### Correlation with ventricular volume

No significant correlation was observed between ventricular volume and the eight metabolites found at lower concentrations in iNPH, whether ventricular volume was measured using the Evans index or MR volumetry (Supplementary Figure 3).

### Association with Alzheimer’s disease pathology in brain biopsies

**Fig. 2:**
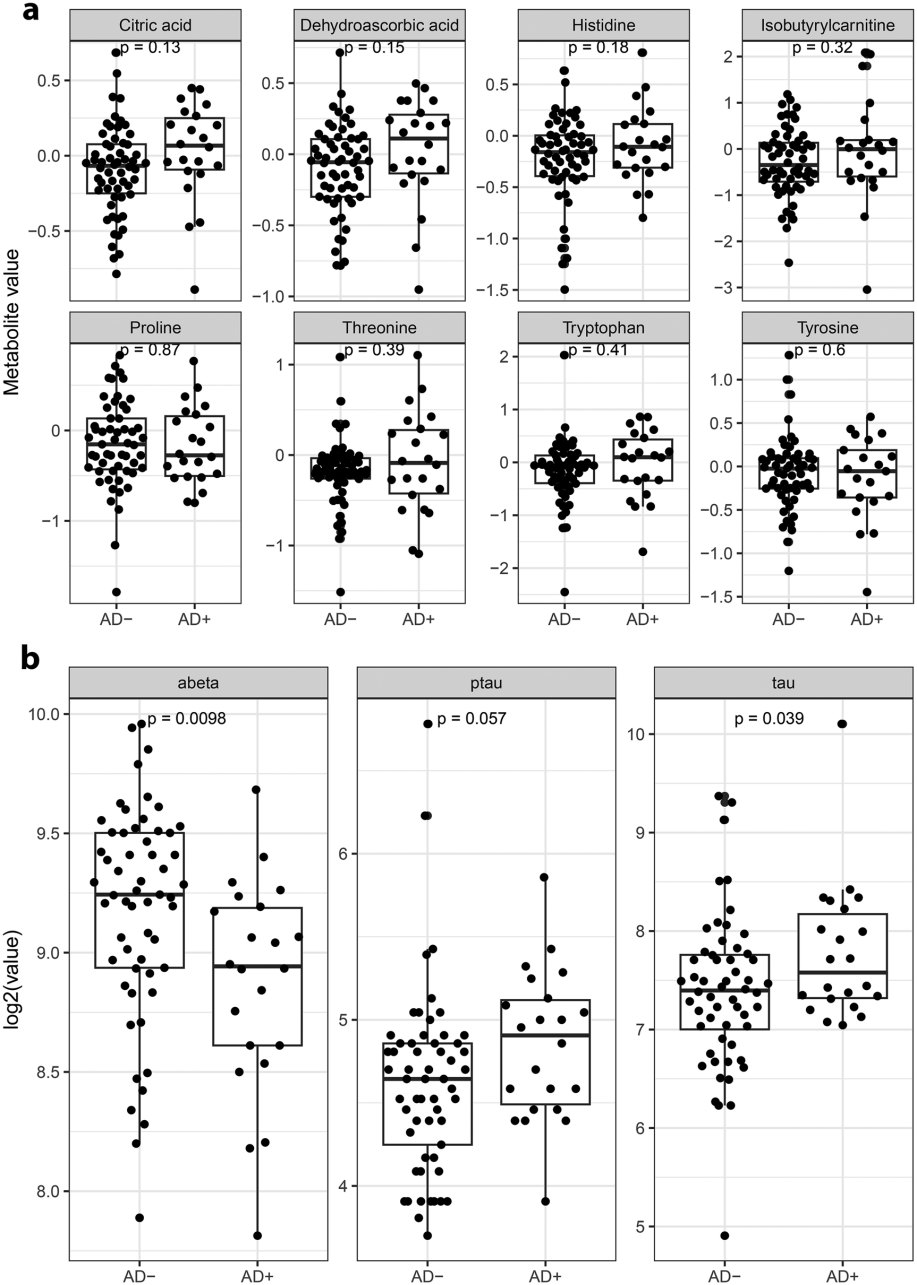
AD biopsies with manually evaluated presence or absence of hyperphosphorylated tau and total amyloid-β aggregations within the biopsies. The association between measured values and AD+/AD was analyzed with a t-test

Cortical brain biopsies were available in 80/120 (66,7%) of patients with iNPH. Among these, 58/80 (73%) were classified as AD+ (presence of both tau and and amyloid-β42). No significant differences in the levels of the eight discriminatory metabolites were observed between patients with brain biopsy findings consistent with AD pathology (AD+) and those without (AD–) (Fig. 2A). In contrast, amyloid-β42 (Aβ42) concentrations were significantly lower (*p* = 0.0098) and total tau levels significantly higher (*p* = 0.039) in the AD+ group (Fig. 2B).

### Distinguishing iNPH using PLS-DA

**Fig. 3:**
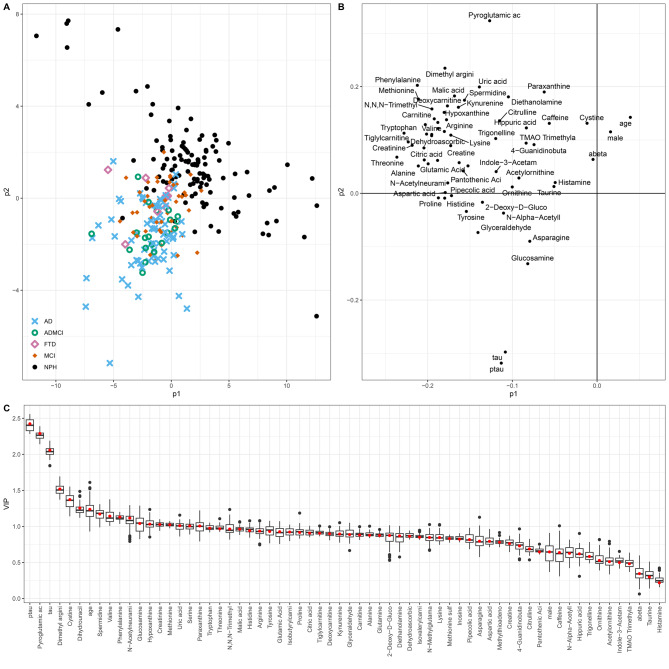
PLS-DA scores and loadings for the model based on age, sex, amyloid-β42, ptau, tau and all metabolites comparing NPH to AD, AD/MCI, MCI, and FTD

Three partial least squares discriminant analysis (PLS-DA) models were constructed to differentiate iNPH from all other groups. The first included demographic variables (age and sex) together with AD CSF biomarkers (Aβ42, total tau, and phosphorylated tau); the second was based solely on the panel of 59 CSF metabolites; and the third integrated all variables. Among these, the integrated model demonstrated the highest discriminative performance, achieving an AUC of 0.97 in distinguishing iNPH from the other diagnostic groups (Table 3, Fig. 3, Supplementary Figures 4–5).

**Table 3:** Performance of PLS-DA models. Mean (standard deviation) of AUC (area under the ROC curve) and error rate (ER) are computed over the 50 test sets. mean (SD)

Variables mean (SD)	AUC	ER
age, sex, amyloid-β42, tau, ptau	0.89 (0.04)	0.19 (0.04)
All 59 metabolites	0.91 (0.04)	0.13 (0.04)
age, sex, amyloid-β42, tau, ptau, all 59 metabolites	0.97 (0.03)	0.08 (0.04)

### Uncertainty-aware prediction for iNPH diagnosis

Although AUC-based evaluation reflects the overall discriminative ability of a model, it does not capture diagnostic uncertainty at the individual level. To address this limitation, we applied conformal prediction (CP), which provides case-level confidence estimates by quantifying uncertainty for each prediction.

The diagnostic performance was assessed by combining the PLS-DA analysis with transductive conformal prediction (TCP). The dataset comprised 118 iNPH cases (82 training, 36 test) and 149 non-iNPH cases (104 training, 45 test), using a 70/30 train–test split.

**Fig. 4:**
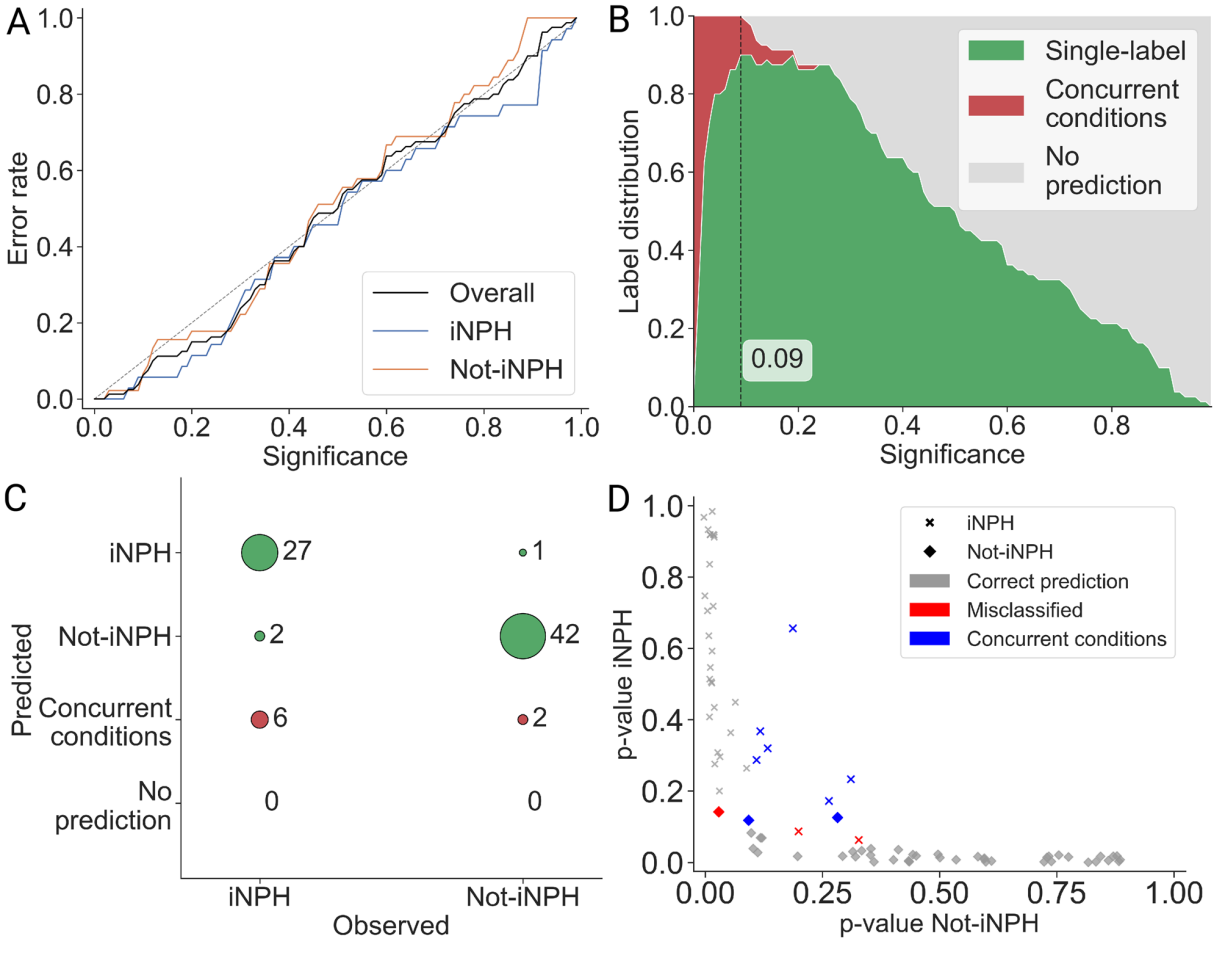
Evaluation of conformal prediction. **A**) calibration curve showing significance levels on the x-axis and observed error rates on the y-axis. The diagonal line represents perfect calibration. **B**) label distribution across significance levels, illustrating the proportion of single-label, multiple-label, and empty-label predictions. The significance level yielding the highest number of single-label predictions was selected for model evaluation (significance level of 0.09). **C**) confusion matrix summarizing predictive performance at the chosen significance level (0.09). The model achieved an accuracy of 94% for iNPH patients and 97% for the not-iNPH group. **D**) predicted p-values for each test individual, with p-values for iNPH on the y-axis and p-values for not-iNPH on the x-axis

The calibration curve demonstrated that TCP yielded a well-calibrated model (Fig. 4a). At a significance level of 0.09 (corresponding to 91% confidence), the model achieved the highest proportion of single-label predictions (Fig. 4b). The corresponding confusion matrix (Fig. 4c) shows that the model confidently and correctly classified 27 of the 35 iNPH cases as single-label predictions. Among the six individuals predicted as having concurrent iNPH, four underwent perioperative cortical biopsy; three were confirmed as AD-positive, supporting the presence of coexisting iNPH and AD spectrum pathology. Figure 4d illustrates the predicted p-values for iNPH (y-axis) and non-iNPH (x-axis), where higher values reflect greater confidence in the corresponding classification, and predictions closer to the origin indicate lower-confidence assignments.

## Discussion

In this study, we applied targeted metabolomic profiling to CSF samples from patients with iNPH, neurodegenerative disorders, and healthy controls. Eight metabolites, five amino acids, one carnitine, one citric acid cycle intermediate, and one vitamin-related compound, were consistently reduced in iNPH compared with all other groups. These alterations were independent of ventricular size and of AD pathology observed in cortical biopsies. A multivariate model integrating metabolite data, demographic variables, and established CSF biomarkers demonstrated excellent diagnostic accuracy in distinguishing iNPH from other conditions. To further enhance clinical applicability, we incorporated transductive conformal prediction, which provided individualized measures of diagnostic uncertainty and improved interpretability at the patient level.

Previous metabolomics studies in iNPH are limited, but differences in the metabolic profiles of AD and iNPH have been reported. These findings particularly included metabolites related to glycerolipid and amino acid metabolism [8]. In the present study, eight metabolites were significantly reduced in the CSF of iNPH patients compared to controls and individuals with MCI, AD, or FTD. One possible interpretation is that these changes reflect a general disturbance in CNS metabolism in iNPH. All analyses were adjusted for age and sex, and a sensitivity analysis using a subset of controls closer in age to the iNPH group confirmed the main findings, although *p*values were somewhat higher due to the smaller sample size. While this suggests that the differences are not solely explained by age, residual confounding cannot be excluded, and we therefore limit mechanistic interpretation to what is directly supported by the data.

Several altered metabolites, including amino acids and intermediates related to energy metabolism and oxidative stress (e.g., citric acid and dehydroascorbic acid), may indicate impaired cellular metabolic activity in the brain parenchyma [25]. However, it remains unclear whether the lower CSF levels are due to reduced production of these metabolites within the brain, possibly due to diminished neuronal or glial function, or altered transport, turnover, or clearance mechanisms, i.e., dysfunction of the glymphatic system. The glymphatic pathway facilitates exchange between interstitial fluid and CSF and is believed to be involved in the clearance of metabolic waste products from the brain [2]. Impaired glymphatic flow could theoretically lead to the accumulation of certain substances in brain tissue or to the reduced appearance of these substances in CSF, depending on the dynamics of transport and clearance. Although glymphatic function is known to decline with age [26–28], the observed metabolite differences are unlikely to be explained solely by aging, as our comparisons included multiple neurodegenerative groups of similar age to the iNPH cohort.

Decreased CSF levels of dehydroascorbic acid, an antioxidant and neurotransmitter precursor, may contribute to oxidative stress and neuroinflammation in iNPH [29]. Citric acid dysregulation could impair neuronal energy metabolism and glymphatic clearance, while altered isobutyrylcarnitine points to mitochondrial β-oxidation deficits [30, 31]. The affected amino acids reflect key neurophysiological roles: proline supports blood–brain barrier integrity and redox balance. Through the kynurenine pathway, tryptophan metabolism may link to inflammation and neurochemical shifts [32, 33]. Tyrosine, a catecholamine precursor, has been associated with ventricular changes, and reduced histidine and threonine may affect neurotransmission and mTOR signaling, potentially influencing cognitive and motor symptoms in iNPH [25, 34, 35].

Previous studies have shown that specific CSF biomarkers in iNPH, such as amyloid precursors, may be lower than in controls and other neurodegenerative disorders [5–7]. It has been suggested that this reduction could be due to a dilution effect related to the enlarged ventricles commonly seen in iNPH [36–38]. Because the eight metabolites distinguishing iNPH patients were consistently lower than in all other groups, we examined whether their concentrations correlated with ventricular volume. No such associations were observed using either the Evans index or MRI-based volumetric measurements. This suggests that the metabolite alterations are not simply a result of ventricular enlargement or CSF dilution, but may instead reflect underlying metabolic changes in the CNS.

Comorbidity with AD is common in iNPH, and previous studies have demonstrated a high prevalence of histopathological AD findings in cortical brain biopsies from iNPH patients [39]. Given that our comparison groups included cohorts representing different stages of AD, we also examined whether AD-related histological findings influenced metabolite levels within the iNPH group. However, no significant differences were observed in any of the eight metabolites between iNPH patients with and without histopathological evidence of AD. Our findings for amyloidβ42 are consistent with the established pattern of lower levels in AD than in other conditions and somewhat lower levels in iNPH than in healthy controls. Withingroup correlations between amyloidβ42, tau, and ptau and age were weak or nonsignificant in most groups (Supplementary Table 1), indicating that age does not account for the observed betweengroup differences. In addition, lower concentrations of tau, phosphorylated tau, and amyloidβ42 in iNPH compared to healthy controls have been consistently reported in previous studies [6, 7, 40], and our findings are in line with this established pattern.

Glymphatic impairment has also been reported in Alzheimer’s disease and other neurodegenerative conditions [41–43], which may contribute to overlapping biomarker profiles and underscores the value of multimodal assessment.

To evaluate the diagnostic utility of the metabolomic data, we also developed PLS-DA models incorporating various combinations of clinical and biochemical variables. A model that integrated all available data, demographics, clinically established CSF biomarkers (abeta, tau, and p-tau), and all 59 identified metabolites showed the highest diagnostic performance. This model achieved substantially better discrimination of iNPH cases than models using only traditional CSF biomarkers or metabolite data alone. These findings highlight the added value of CSF metabolomics in improving diagnostic accuracy and suggest that incorporating metabolic profiling could have a potential role in future diagnostic workflows of iNPH.

In addition, integrating TCP with PLS-DA enhanced the diagnostic utility of our approach by introducing an uncertainty-aware framework. At a prediction confidence of 91%, the model confidently identified 27 of the 35 iNPH patients as single-label predictions, underscoring its reliability in classifying the majority of true iNPH cases. Six iNPH patients in the test set were flagged as having concurrent conditions, reflecting overlap with the not-iNPH group and suggesting the presence of AD spectrum pathology. The model misclassified two iNPH and one not-iNPH patient; however, increasing the confidence threshold highlighted these cases as potential concurrent conditions rather than simple misclassifications. This underscores the value of incorporating uncertainty, as higher confidence thresholds identify additional patients who may benefit from closer monitoring [44]. Such patients should be considered for further diagnostic evaluation before treatment decisions are made.

### Strengths

This study benefits from using a well-characterized, single-center cohort, ensuring clinical assessment and sample handling consistency. The inclusion of multiple diagnostic groups allows for relevant differential comparisons and increases the clinical relevance of the findings. A notable strength is the use of HPLC-HRMS, which enables broad, sensitive, and reproducible detection of metabolites across a wide dynamic range. Targeted assays, pooled quality control samples, and statistical adjustments for potential confounders enhance the reliability and comparability of the metabolomic data.

### Limitations

One limitation of this study is the difference in age distribution between the control group and other diagnostic groups. Although all analyses were adjusted for age and sex, and a sensitivity analysis using age-matched controls confirmed the main findings, residual confounding cannot be excluded. This should be considered when interpreting the results. Also, the sample size in some diagnostic subgroups, such as FTD, was small.

The cross-sectional design limits conclusions about longitudinal changes or treatment effects. Furthermore, the chromatographic method used was optimized for semi-polar to non-polar metabolites. Analysis of highly polar metabolites would require a different LC-HRMS approach, such as HILIC, which we plan for future studies. Confirmational studies on separate cohorts are also needed to be more certain about how generalizable the results are.

Finally, we did not assess potential differences between ventricular and lumbar CSF metabolomic profiles. To our knowledge, no studies have directly compared these compartments, and future research should investigate whether such differences could influence biomarker interpretation.

## Conclusion

This study identifies a distinct CSF metabolite profile that differentiates iNPH from other neurodegenerative disorders, including AD, MCI, and FTD. Eight metabolites related to neurotransmission, oxidative stress, and energy metabolism were consistently reduced in iNPH, independent of ventricular size or AD pathology. The strong performance of a multivariate model combining metabolomic, demographic, and established biomarker data underscores the diagnostic potential of this approach, and CP extends this by providing patient-specific confidence estimates. These findings support the integration of CSF metabolomics into diagnostic workflows to improve accuracy and guide clinical decision-making in iNPH.

## Electronic supplementary material

Below is the link to the electronic supplementary material.

Supplementary Material 1

## Data Availability

De-identified data that support the findings of this study are available from the corresponding author upon reasonable request.
