# Supplementary material for: Targeted CSF metabolomics and conformal prediction improve diagnostic accuracy of normal pressure hydrocephalus
Source: Fluids Barriers CNS. 2026 Feb 7;23:34. doi: 10.1186/s12987-026-00771-z (PMC12930833; doi:10.1186/s12987-026-00771-z)
Supplement: Supplementary file 1 — Supplementary Material 1 [file 12987_2026_771_MOESM1_ESM.pdf]

SUPPLEMENTARY TABLES

Supplementary Table 1**.** *Correlation of biomarkers with age within each respective group.*

Group	r	p
Amyloid-beta 42		
**AD**	-0.01471060	0.903089049
**ADMCI**	0.18560589	0.408251966
**C**	-0.04932793	0.861411996
**FTD**	0.40719293	0.316709185
**MCI**	-0.40650687	0.004142931
**iNPH**	0.13838401	0.135062492
**ULSAM**	0.03840440	0.849166335
Phosphorylated tau		
**AD**	-0.07906133	0.512219975
**ADMCI**	0.25248068	0.256962614
**C**	0.24115875	0.386567446
**FTD**	-0.03592879	0.932691475
**MCI**	0.11730311	0.427181368
**iNPH**	0.26232919	0.004108276
**ULSAM**	-0.16019131	0.424774241
Total tau		
**AD**	-0.07136508	0.554243028
**ADMCI**	0.24964700	0.262520348
**C**	0.23385091	0.401552695

**FTD**	-0.03592879	0.932691475
**MCI**	0.24209745	0.097347341
**iNPH**	0.27621794	0.002573589
**ULSAM**	-0.13701028	0.495578251

Abbreviations: AD= Alzheimers Disease, ADMCI= Alzheimer's Disease–related Mild Cognitive Impairment, C= Controls, FTD= Frontotemporal Dementia, MCI= Mild Cognitive Impairment, iNPH ULSAM = the subgroup of healthy individuals recruited from Uppsala longitudinal study of adult men.

Supplementary Table 2**.** *59 metabolites were semi quantified in CSF from 383 samples. Linear regression was performed for one metabolite at the time with metabolite as dependent variable and adjusted for age and sex. ANOVA F-test was used to identify metabolites that differ significantly between groups.*

**Metabolite**	**p.anova**	**q.anova**	**contrast**	**log2FC**	**p**
Isobutyrylcarnitine	2,00E-07	1,09E-05	NPH - AD	-4,81E-01	1,16E-05
			NPH - ADMCI	-5,46E-01	6,83E-04
			NPH - MCI	-3,73E-01	3,49E-03
			NPH - FTD	-1,12E+00	2,18E-05
			NPH - C	-4,32E-01	2,76E-03
Proline	4,79E-07	1,09E-05	NPH - AD	-4,03E-01	2,91E-07
			NPH - ADMCI	-2,91E-01	9,97E-03
			NPH - MCI	-3,47E-01	1,44E-04
			NPH - FTD	-5,91E-01	1,33E-03
			NPH - C	-2,76E-01	6,78E-03
Threonine	5,54E-07	1,09E-05	NPH - AD	-2,78E-01	3,84E-07

			NPH - ADMCI	-3,24E-01	5,40E-05
			NPH - MCI	-1,65E-01	9,29E-03
			NPH - FTD	-2,65E-01	4,01E-02
			NPH - C	-2,14E-01	2,83E-03
Histidine	8,40E-07	1,24E-05	NPH - AD	-2,42E-01	5,31E-05
			NPH - ADMCI	-3,03E-01	5,56E-04
			NPH - MCI	-3,40E-01	1,49E-06
			NPH - FTD	-2,79E-01	4,96E-02
			NPH - C	-2,17E-01	5,98E-03
Tyrosine	1,96E-06	2,31E-05	NPH - AD	-2,90E-01	9,18E-05
			NPH - ADMCI	-3,08E-01	4,33E-03
			NPH - MCI	-4,19E-01	1,60E-06
			NPH - FTD	-3,91E-01	2,57E-02
			NPH - C	-2,71E-01	5,77E-03
Tryptophan	2,48E-06	2,44E-05	NPH - AD	-3,05E-01	4,70E-05
			NPH - ADMCI	-4,69E-01	2,25E-05
			NPH - MCI	-3,20E-01	2,65E-04
			NPH - FTD	-4,60E-01	9,87E-03
			NPH - C	-2,25E-01	2,22E-02
Citric acid	3,17E-06	2,67E-05	NPH - AD	-2,09E-01	2,52E-06
			NPH - ADMCI	-1,83E-01	4,73E-03
			NPH - MCI	-1,59E-01	2,11E-03
			NPH - FTD	-3,34E-01	1,58E-03
			NPH - C	-1,59E-01	6,48E-03
Pipecolic acid	4,27E-06	3,15E-05	NPH - AD	-3,16E-01	4,17E-06
			NPH - ADMCI	-3,46E-01	5,53E-04

			NPH - MCI	-2,44E-01	2,19E-03
			NPH - FTD	-7,02E-02	6,64E-01
			NPH - C	-2,95E-01	1,10E-03
Alanine	6,82E-06	4,47E-05	NPH - AD	-1,98E-01	1,48E-04
			NPH - ADMCI	-1,42E-01	6,40E-02
			NPH - MCI	-1,66E-01	6,56E-03
			NPH - FTD	-2,90E-01	1,99E-02
			NPH - C	-3,03E-01	1,36E-05

Tiglylcarnitine;C12H21NO4;	2,95E-05	1,74E-04	NPH - AD	-4,05E-01	3,89E-06
			NPH - ADMCI	-3,16E-01	1,33E-02
			NPH - MCI	-2,98E-01	3,44E-03
			NPH - FTD	-6,04E-01	3,69E-03
			NPH - C	-1,90E-01	9,65E-02
Dehydroascorbic acid	7,98E-05	4,28E-04	NPH - AD	-1,89E-01	4,60E-05
			NPH - ADMCI	-1,75E-01	1,00E-02
			NPH - MCI	-1,52E-01	4,95E-03
			NPH - FTD	-2,89E-01	9,04E-03
			NPH - C	-1,62E-01	7,95E-03
Glucosamine	1,00E-04	4,92E-04	NPH - AD	-3,27E-01	6,84E-05
			NPH - ADMCI	-1,89E-01	1,15E-01
			NPH - MCI	-2,38E-01	1,26E-02
			NPH - FTD	-4,08E-01	3,63E-02
			NPH - C	-3,70E-01	6,40E-04
Pyroglutamic acid	2,26E-04	1,02E-03	NPH - AD	1,80E-01	1,38E-03
			NPH - ADMCI	1,47E-01	7,37E-02
			NPH - MCI	2,39E-01	2,99E-04
			NPH - FTD	8,63E-02	5,19E-01
			NPH - C	2,37E-01	1,48E-03
Aspartic acid	7,07E-04	2,98E-03	NPH - AD	-2,76E-01	2,42E-04
			NPH - ADMCI	-3,33E-01	2,59E-03
			NPH - MCI	-2,19E-01	1,29E-02
			NPH - FTD	-1,89E-01	2,92E-01
			NPH - C	-2,45E-01	1,38E-02
Glyceraldehyde	8,64E-04	3,40E-03	NPH - AD	-2,66E-01	5,45E-05

			NPH - ADMCI	-2,40E-01	1,26E-02
			NPH - MCI	-2,19E-01	4,41E-03
			NPH - FTD	-1,53E-01	3,26E-01
			NPH - C	-1,50E-01	8,31E-02
Isovalerylcarnitine	1,56E-03	5,77E-03	NPH - AD	-3,47E-01	2,02E-04
			NPH - ADMCI	-2,70E-01	4,81E-02
			NPH - MCI	-1,95E-01	7,32E-02
			NPH - FTD	-5,88E-01	8,32E-03
			NPH - C	-2,08E-01	9,09E-02
Asparagine	1,83E-03	6,36E-03	NPH - AD	-3,24E-01	3,61E-03
			NPH - ADMCI	-4,06E-01	1,32E-02
			NPH - MCI	-3,60E-01	5,84E-03
			NPH - FTD	-2,87E-01	2,79E-01
			NPH - C	-4,29E-01	3,63E-03
Ornithine	8,50E-03	2,67E-02	NPH - AD	-2,97E-01	1,34E-02
			NPH - ADMCI	-5,09E-01	3,87E-03
			NPH - MCI	-2,63E-01	6,11E-02
			NPH - FTD	-5,75E-01	4,42E-02
			NPH - C	-3,07E-01	5,26E-02
Glutamine	8,58E-03	2,67E-02	NPH - AD	-1,18E-01	1,37E-03
			NPH - ADMCI	-6,54E-02	2,27E-01
			NPH - MCI	-1,12E-01	9,60E-03
			NPH - FTD	-1,71E-01	5,25E-02
			NPH - C	-8,44E-02	8,37E-02
Methionine sulfoxide	9,28E-03	2,74E-02	NPH - AD	-1,78E-01	2,93E-03
			NPH - ADMCI	-2,25E-01	1,04E-02

			NPH - MCI	-1,62E-01	2,05E-02
			NPH - FTD	-1,95E-01	1,70E-01
			NPH - C	-1,52E-01	5,40E-02
Methionine	1,28E-02	3,55E-02	NPH - AD	-2,12E-01	1,21E-03
			NPH - ADMCI	-2,25E-01	1,91E-02
			NPH - MCI	-1,43E-01	6,24E-02
			NPH - FTD	-2,66E-01	8,88E-02
			NPH - C	-1,14E-01	1,85E-01
N-Alpha-Acetyllysine	1,32E-02	3,55E-02	NPH - AD	-3,14E-01	5,14E-04
			NPH - ADMCI	-1,27E-01	3,45E-01
			NPH - MCI	-2,08E-01	4,87E-02
			NPH - FTD	-1,52E-01	4,70E-01
			NPH - C	-2,32E-01	5,02E-02
Histamine	1,56E-02	4,00E-02	NPH - AD	2,56E-02	9,05E-01
			NPH - ADMCI	-1,41E-01	6,57E-01
			NPH - MCI	-2,84E-01	2,61E-01
			NPH - FTD	5,28E-01	3,07E-01
			NPH - C	-9,36E-01	1,21E-03
Inosine	1,77E-02	4,26E-02	NPH - AD	-2,31E-01	1,69E-03
			NPH - ADMCI	-1,86E-01	8,43E-02
			NPH - MCI	-1,04E-01	2,27E-01
			NPH - FTD	-3,71E-01	3,50E-02
			NPH - C	-1,40E-01	1,48E-01
Methylthioadenosine	1,81E-02	4,26E-02	NPH - AD	-2,18E-01	2,30E-03
			NPH - ADMCI	-4,65E-02	6,56E-01
			NPH - MCI	-1,50E-01	7,12E-02

			NPH - FTD	-2,46E-01	1,49E-01
			NPH - C	-2,04E-01	3,07E-02
Pantothenic Acid	2,26E-02	5,12E-02	NPH - AD	-2,03E-01	5,01E-02
			NPH - ADMCI	-4,34E-01	4,39E-03
			NPH - MCI	-2,70E-01	2,65E-02
			NPH - FTD	-1,32E-01	5,91E-01
			NPH - C	-2,60E-01	5,75E-02
Deoxycarnitine	2,49E-02	5,44E-02	NPH - AD	-2,27E-01	3,52E-03
			NPH - ADMCI	-1,47E-02	8,97E-01
			NPH - MCI	-1,74E-01	5,61E-02
			NPH - FTD	-3,19E-01	8,61E-02
			NPH - C	-1,69E-01	1,01E-01
Spermidine	2,97E-02	6,27E-02	NPH - AD	-1,84E-01	9,22E-02
			NPH - ADMCI	2,31E-01	1,50E-01
			NPH - MCI	2,00E-01	1,20E-01
			NPH - FTD	-3,29E-01	2,09E-01
			NPH - C	6,83E-03	9,62E-01
Cystine	3,26E-02	6,64E-02	NPH - AD	4,94E-01	1,51E-03
			NPH - ADMCI	3,05E-01	1,79E-01
			NPH - MCI	3,34E-01	6,58E-02
			NPH - FTD	3,84E-01	2,98E-01
			NPH - C	3,55E-01	8,31E-02
Phenylalanine	4,95E-02	9,54E-02	NPH - AD	-2,04E-01	4,23E-03
			NPH - ADMCI	-1,85E-01	7,63E-02
			NPH - MCI	-1,61E-01	5,26E-02
			NPH - FTD	-2,77E-01	1,03E-01

			NPH - C	-8,44E-02	3,73E-01
Creatinine	5,03E-02	9,54E-02	NPH - AD	-1,82E-01	2,43E-03
			NPH - ADMCI	-1,05E-01	2,33E-01
			NPH - MCI	-1,02E-01	1,48E-01
			NPH - FTD	-2,12E-01	1,39E-01
			NPH - C	-1,13E-01	1,53E-01
Serine	5,18E-02	9,54E-02	NPH - AD	-1,44E-01	1,40E-02
			NPH - ADMCI	-1,86E-01	3,05E-02
			NPH - MCI	-1,07E-01	1,17E-01
			NPH - FTD	-1,55E-01	2,67E-01
			NPH - C	-1,53E-01	4,80E-02
Carnitine	5,86E-02	1,05E-01	NPH - AD	-2,10E-01	7,02E-03
			NPH - ADMCI	-1,18E-01	3,04E-01
			NPH - MCI	-1,74E-01	5,66E-02
			NPH - FTD	-3,12E-01	9,43E-02
			NPH - C	-1,59E-01	1,22E-01
Creatine	8,46E-02	1,47E-01	NPH - AD	-1,02E-01	6,68E-02
			NPH - ADMCI	-3,15E-02	6,98E-01
			NPH - MCI	-5,38E-02	4,06E-01
			NPH - FTD	-1,49E-01	2,60E-01
			NPH - C	-1,97E-01	7,50E-03
Hippuric acid	1,16E-01	1,95E-01	NPH - AD	-1,57E-01	3,90E-01
			NPH - ADMCI	-1,19E-01	6,58E-01
			NPH - MCI	-2,85E-01	1,84E-01
			NPH - FTD	-8,41E-01	5,58E-02
			NPH - C	4,20E-01	8,39E-02

Acetylornithine	1,28E-01	2,10E-01	NPH - AD	-3,54E-01	5,42E-02
			NPH - ADMCI	-4,83E-01	7,40E-02
			NPH - MCI	-4,99E-01	2,08E-02
			NPH - FTD	-3,50E-01	4,26E-01
			NPH - C	-3,00E-01	2,18E-01
N,N,N-Trimethyllysine	1,44E-01	2,30E-01	NPH - AD	-1,66E-01	3,41E-02
			NPH - ADMCI	-2,66E-01	2,08E-02
			NPH - MCI	-8,89E-02	3,32E-01
			NPH - FTD	-9,72E-02	6,03E-01
			NPH - C	-1,25E-01	2,26E-01
N-Acetylneuraminate	1,60E-01	2,48E-01	NPH - AD	-1,70E-01	3,06E-02
			NPH - ADMCI	-2,24E-01	5,31E-02
			NPH - MCI	-7,92E-02	3,89E-01
			NPH - FTD	-1,06E-01	5,72E-01
			NPH - C	-1,69E-01	1,04E-01
Citrulline	1,73E-01	2,61E-01	NPH - AD	-2,97E-02	7,37E-01
			NPH - ADMCI	-2,13E-02	8,70E-01
			NPH - MCI	-2,49E-01	1,71E-02
			NPH - FTD	-3,23E-01	1,29E-01
			NPH - C	-5,01E-02	6,69E-01
Lysine	1,80E-01	2,62E-01	NPH - AD	-9,14E-02	8,33E-02
			NPH - ADMCI	-1,75E-01	2,49E-02
			NPH - MCI	-1,07E-01	8,33E-02
			NPH - FTD	-1,96E-02	8,77E-01
			NPH - C	-6,23E-02	3,73E-01
Kynurenine	1,82E-01	2,62E-01	NPH - AD	-1,80E-01	6,47E-02

			NPH - ADMCI	-5,87E-02	6,81E-01
			NPH - MCI	-2,39E-01	3,67E-02
			NPH - FTD	-3,90E-01	9,45E-02
			NPH - C	-8,49E-02	5,10E-01
Diethanolamine	2,00E-01	2,81E-01	NPH - AD	-1,08E-01	4,58E-01
			NPH - ADMCI	-2,05E-01	3,42E-01
			NPH - MCI	-2,61E-01	1,29E-01
			NPH - FTD	-3,92E-01	2,63E-01
			NPH - C	3,27E-01	9,17E-02
Dihydrouracil	2,12E-01	2,90E-01	NPH - AD	-9,73E-02	9,13E-02
			NPH - ADMCI	-5,09E-02	5,48E-01
			NPH - MCI	4,07E-03	9,52E-01
			NPH - FTD	-2,91E-01	3,57E-02
			NPH - C	-4,05E-02	5,95E-01
Hypoxanthine	2,40E-01	3,22E-01	NPH - AD	-9,56E-02	2,26E-01
			NPH - ADMCI	-1,31E-01	2,61E-01
			NPH - MCI	-9,81E-02	2,91E-01
			NPH - FTD	2,55E-01	1,78E-01
			NPH - C	-1,49E-01	1,54E-01
2-Deoxy-D-Glucose	2,59E-01	3,39E-01	NPH - AD	-2,75E-01	3,98E-02
			NPH - ADMCI	-5,26E-02	7,89E-01
			NPH - MCI	-1,85E-01	2,38E-01
			NPH - FTD	-5,49E-01	8,66E-02
			NPH - C	-8,18E-02	6,45E-01
Valine	2,66E-01	3,41E-01	NPH - AD	-9,34E-02	9,11E-02
			NPH - ADMCI	2,12E-03	9,79E-01

			NPH - MCI	-8,95E-02	1,68E-01
			NPH - FTD	-2,39E-01	7,17E-02
			NPH - C	8,35E-03	9,09E-01
Indole-3-Acetamide	2,86E-01	3,59E-01	NPH - AD	-2,22E-01	1,15E-01
			NPH - ADMCI	-2,90E-01	1,59E-01
			NPH - MCI	-1,95E-01	2,36E-01
			NPH - FTD	-3,98E-01	2,36E-01
			NPH - C	-3,16E-01	9,27E-02
Malic acid	2,95E-01	3,62E-01	NPH - AD	-1,65E-01	4,32E-02
			NPH - ADMCI	-2,08E-01	8,29E-02
			NPH - MCI	-1,30E-01	1,73E-01
			NPH - FTD	-1,23E-01	5,30E-01
			NPH - C	-9,81E-02	3,64E-01
Paraxanthine	3,17E-01	3,82E-01	NPH - AD	3,70E-01	6,26E-02
			NPH - ADMCI	1,47E-02	9,60E-01
			NPH - MCI	1,20E-01	6,07E-01
			NPH - FTD	1,32E-02	9,78E-01
			NPH - C	4,53E-01	8,52E-02
N-Methylglutamate	3,76E-01	4,44E-01	NPH - AD	-1,17E-01	1,48E-01
			NPH - ADMCI	-4,92E-02	6,78E-01
			NPH - MCI	-8,35E-02	3,77E-01
			NPH - FTD	-2,74E-01	1,56E-01
			NPH - C	-1,71E-01	1,10E-01
Glutamic Acid	4,07E-01	4,67E-01	NPH - AD	-5,97E-02	4,36E-01
			NPH - ADMCI	-1,79E-01	1,13E-01
			NPH - MCI	-1,15E-01	1,99E-01

			NPH - FTD	-9,84E-02	5,92E-01
			NPH - C	-1,55E-01	1,29E-01
Trigonelline	4,15E-01	4,67E-01	NPH - AD	-1,87E-01	1,67E-01
			NPH - ADMCI	-2,64E-01	1,85E-01
			NPH - MCI	-2,24E-01	1,59E-01
			NPH - FTD	-4,86E-01	1,35E-01
			NPH - C	-2,47E-02	8,91E-01
Arginine	4,24E-01	4,67E-01	NPH - AD	-8,06E-02	1,40E-01
			NPH - ADMCI	-4,90E-02	5,43E-01
			NPH - MCI	-7,65E-02	2,33E-01
			NPH - FTD	5,51E-02	6,74E-01
			NPH - C	-1,11E-01	1,28E-01
4-Guanidinobutanoate	4,27E-01	4,67E-01	NPH - AD	3,26E-02	6,45E-01
			NPH - ADMCI	1,10E-01	2,93E-01
			NPH - MCI	-1,19E-03	9,89E-01
			NPH - FTD	-1,18E-01	4,88E-01
			NPH - C	1,72E-01	6,74E-02
Uric acid	4,66E-01	5,00E-01	NPH - AD	-1,50E-01	1,43E-01
			NPH - ADMCI	-1,37E-01	3,62E-01
			NPH - MCI	-1,15E-01	3,37E-01
			NPH - FTD	-3,34E-01	1,74E-01
			NPH - C	7,37E-02	5,87E-01
Dimethyl arginine (sum of Symmetric and Asymmetric)	5,09E-01	5,36E-01	NPH - AD	-3,95E-02	5,26E-01
			NPH - ADMCI	2,72E-02	7,67E-01
			NPH - MCI	1,11E-01	1,30E-01
			NPH - FTD	-6,22E-02	6,78E-01

			NPH - C	2,54E-02	7,59E-01
TMAO Trimethylamine-N-Oxide	5,25E-01	5,43E-01	NPH - AD	-1,95E-01	2,23E-01
			NPH - ADMCI	-1,39E-01	5,56E-01
			NPH - MCI	-1,89E-01	3,17E-01
			NPH - FTD	-6,64E-01	8,49E-02
			NPH - C	-1,27E-02	9,52E-01
Caffeine	5,78E-01	5,88E-01	NPH - AD	3,40E-01	1,67E-01
			NPH - ADMCI	-5,94E-02	8,69E-01
			NPH - MCI	-1,85E-01	5,20E-01
			NPH - FTD	-2,41E-01	6,82E-01
			NPH - C	1,47E-01	6,50E-01
Taurine	9,65E-01	9,65E-01	NPH - AD	4,28E-02	7,58E-01
			NPH - ADMCI	-8,56E-02	6,67E-01
			NPH - MCI	-7,54E-02	6,36E-01
			NPH - FTD	-2,81E-02	9,34E-01
			NPH - C	-9,86E-02	5,85E-01

SUPPLEMENTARY FIGURES

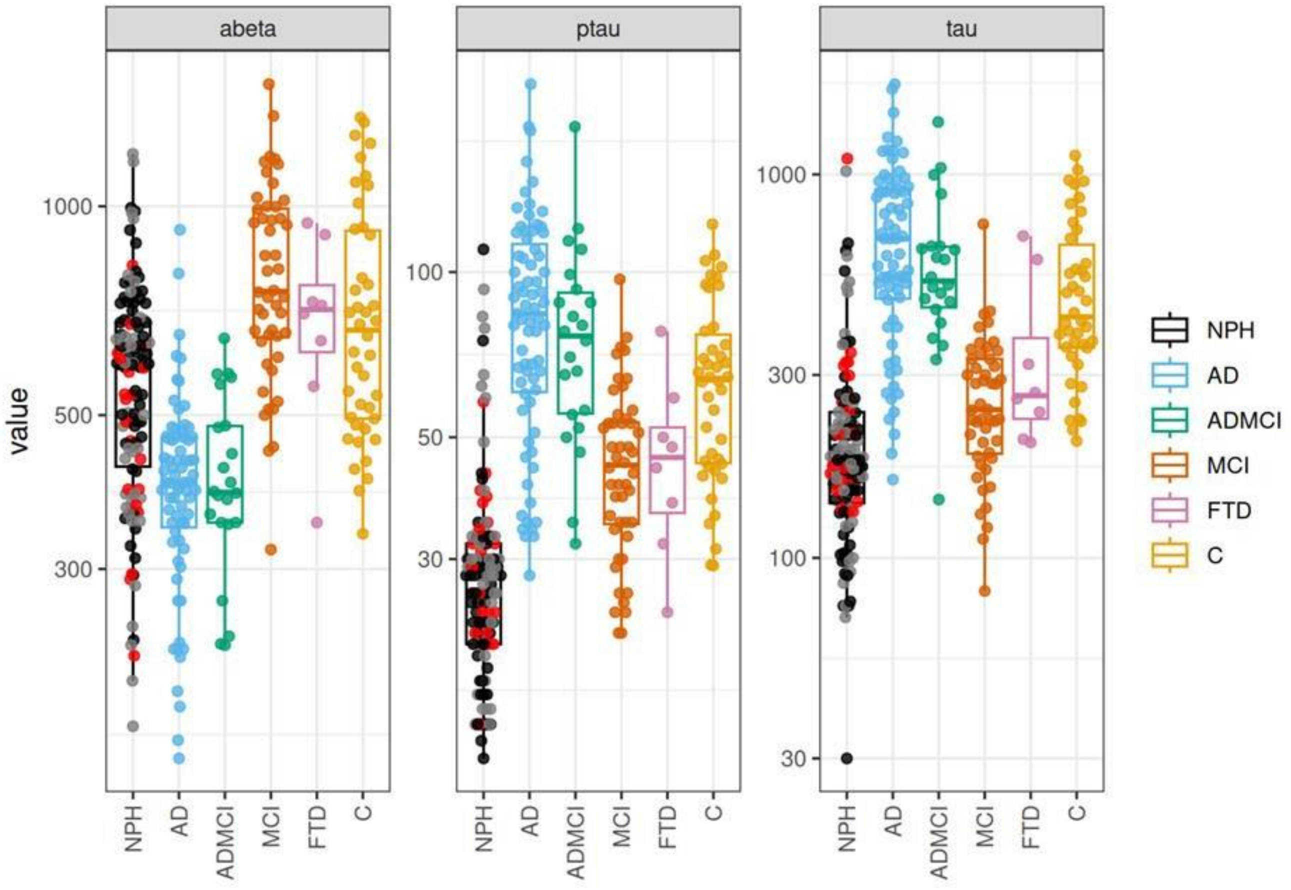

Supplementary *Figure 1. Summary of CSF measurement of abeta, ptau and tau. For NPH the AD+ are marked red and AD- are marked black and patients with no biopsy are grey.*

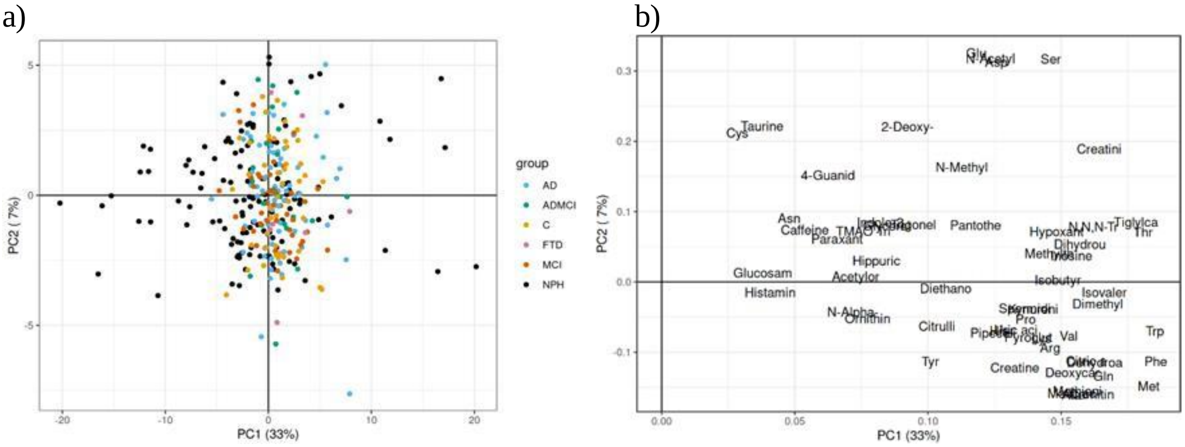

Supplementary Figure 2. *Principal component analysis (PCA) with score plot colored according to patient groups. All included data is reduced into two dimensions. PC1 is the combination with the largest possible explained variation, PC2 is the second most important direction and ortogonal from PC1. Loading plot illustrated in (B).*

a)

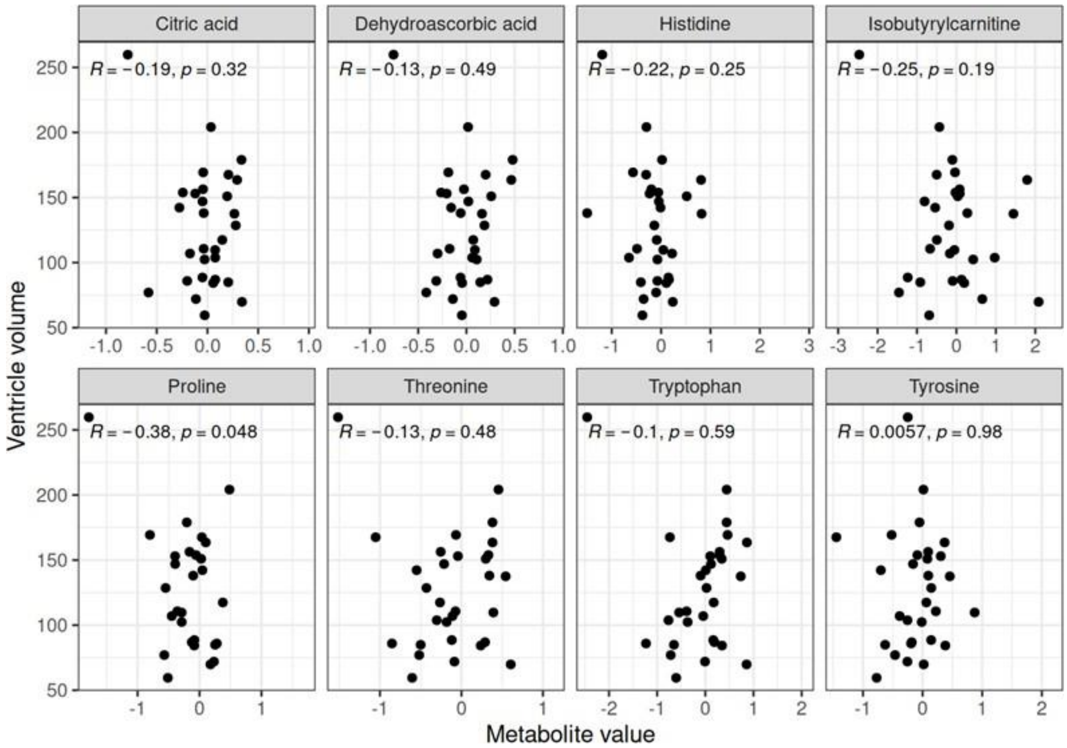

b)

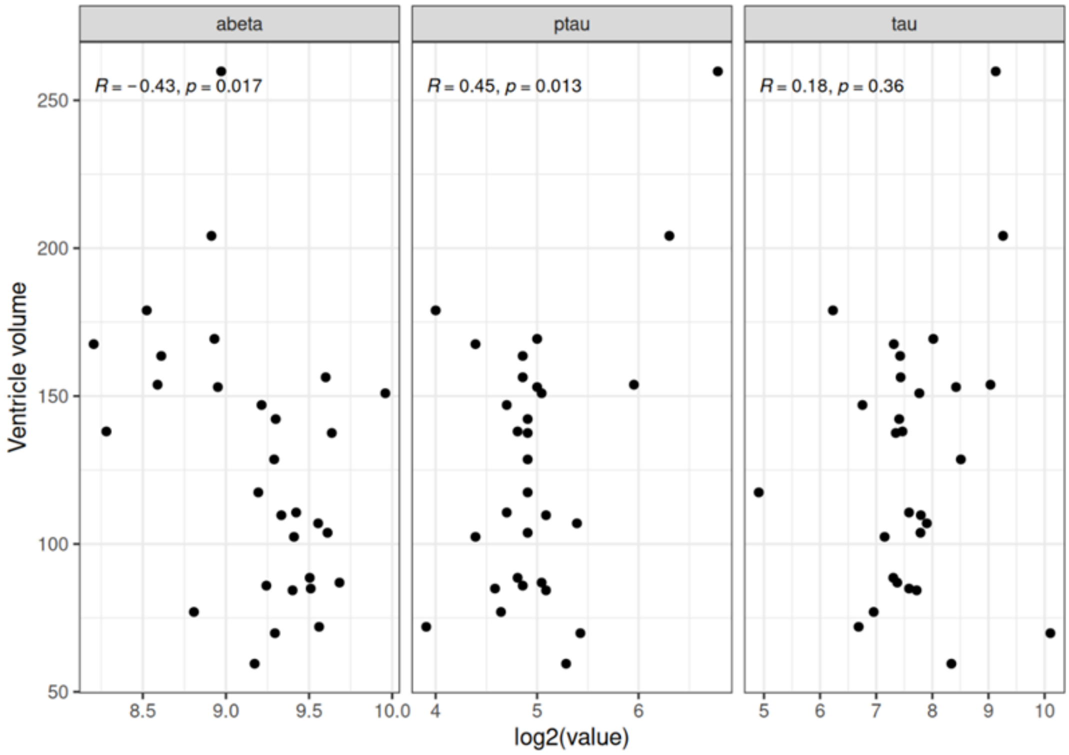

Supplementary Figure 3. *Correlation between measured values and ventricle volume. Correlation is computed using Pearson's correlation.*

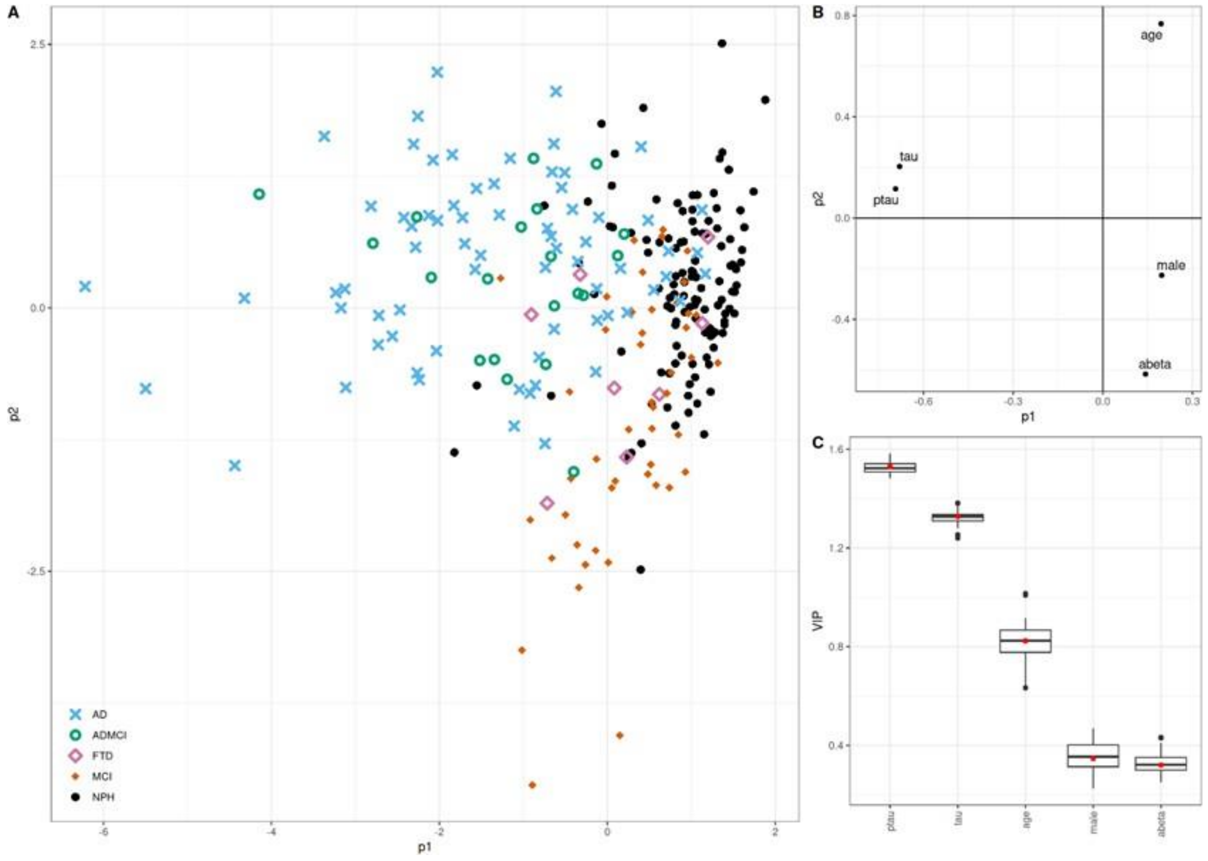

Supplementary Figure 4. *PLS-DA scores, loadings and VIP plot for the model based on age, sex, abeta, tau and ptau comparing NPH to AD, AD/MCI, MCI, and FTD. The plot shows results for a model trained and evaluated on all samples. The VIP (variable importance) values for clinical and biomarker variables in the PLS-DA model predicting patient or control spinal samples based on PEA data indicate how important each variable is to the model. The red dots represent the VIP for the full model (based on all data). The boxplots represent the VIP for the cross validated models.*

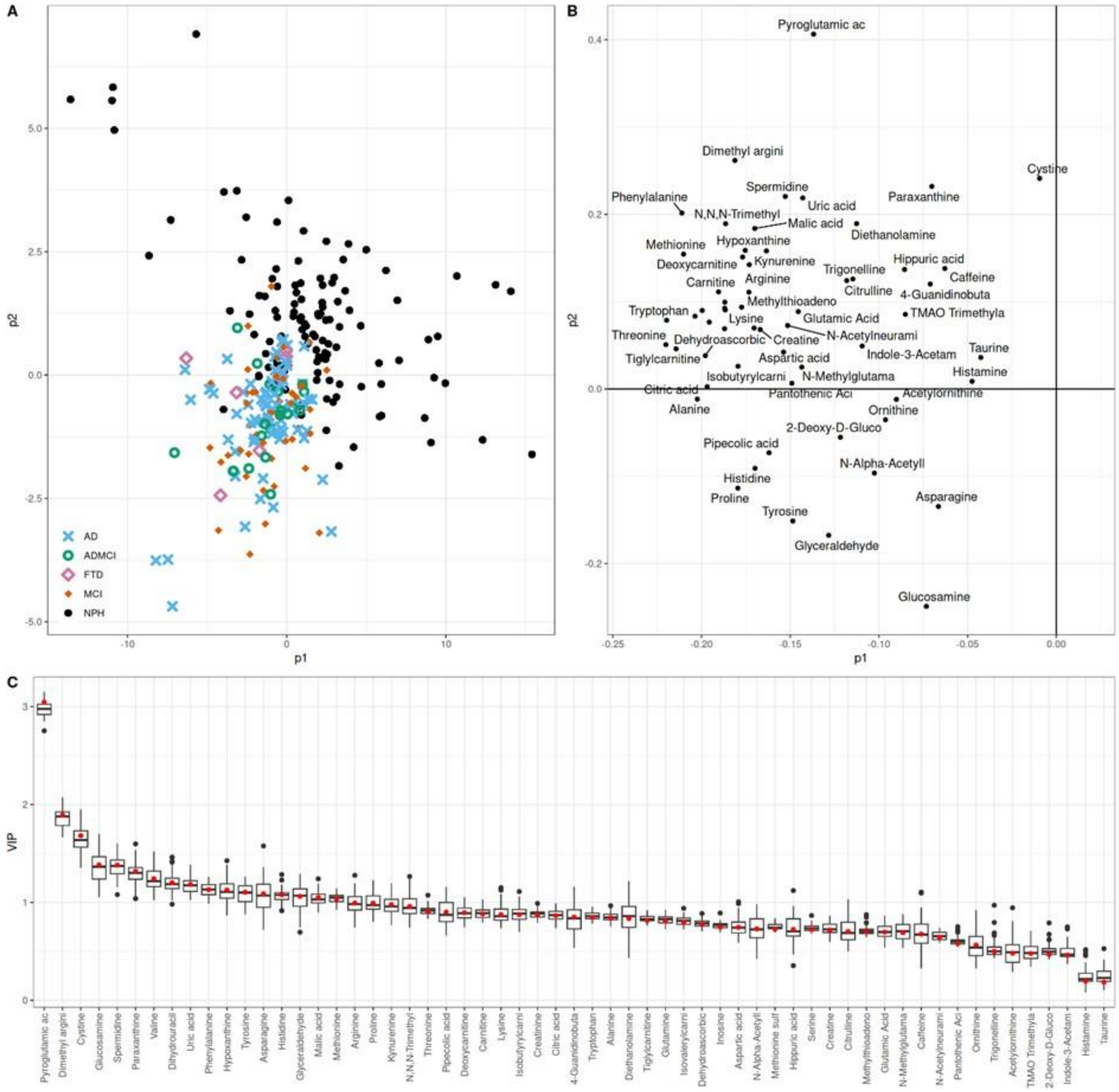

Supplementary Figure 5. *PLS-DA scores, loadings and VIP plot for the model based on all metabolites comparing NPH to AD, AD/MCI, MCI, and FTD. The plot shows results for a model trained and evaluated on all samples. The VIP (variable importance) values for clinical and biomarker variables in the PLS-DA model predicting patient or control spinal samples based on PEA data indicate how important each variable is to the model. The red dots represent the VIP for the full model (based on all data). The boxplots represent the VIP for the cross validated models.*
